# Dilation Device Use and Concomitant Antegrade Stenting are Associated With Procedure‐related Early Adverse Events After Endoscopic Ultrasound‐guided Hepaticogastrostomy: A Retrospective Multicenter Study

**DOI:** 10.1002/deo2.70211

**Published:** 2025-10-07

**Authors:** Shinichi Hashimoto, Hiroki Taguchi, Norimasa Araki, Yu Yamazato, Hiroki Iwata, Yuji Tabira, Ryusuke Shibata, Yusuke Kamikihara, Koshiro Toyodome, Issei Kojima, Takafumi Hamada, Kengo Tsuneyoshi, Yoshitaka Nakamura, Hiroki Yano, Makoto Hinokuchi, Shiho Arima, Shiroh Tanoue, Fumisato Sasaki, Shuji Kanmura, Akio Ido

**Affiliations:** ^1^ Digestive and Lifestyle Diseases, Kagoshima University Graduate School of Medical and Dental Sciences Kagoshima Japan; ^2^ Department of Gastroenterology Kagoshima City Hospital Kagoshima Japan; ^3^ Department of Gastroenterology Izumi General Medical Center Kagoshima Japan; ^4^ Department of Gastroenterology Kagoshima Kouseiren Hospital Kagoshima Japan; ^5^ Department of Gastroenterology Imamura General Hospital Kagoshima Japan; ^6^ Department of Gastroenterology Saiseikai Sendai Hospital Kagoshima Japan

**Keywords:** acute peritonitis, antegrade stenting, endoscopic ultrasound‐guided hepaticogastrostomy, fistula dilation, procedure‐related early adverse event

## Abstract

**Objectives:**

Endoscopic ultrasound‐guided hepaticogastrostomy (EUS‐HGS) is useful in cases of endoscopic retrograde cholangiopancreatography failure. However, the procedure has a high incidence of procedure‐related early adverse events (PRAEs). This study retrospectively evaluated risk factors for such events post‐EUS‐HGS.

**Methods:**

This multicenter study included 222 patients (120 males and 102 females; median age = 73 years) who underwent initial EUS‐HGS. The clinical success rate and PRAE incidence, and risk factors were analyzed. PRAEs were defined as AEs occurring within 2 weeks.

**Results:**

The median procedure time was 41 min. Metal or plastic stents were used for EUS‐HGS in 107 and 115 patients, respectively. Fistula dilation and concomitant antegrade stenting (AGS) were performed in 166 and 45 patients, respectively. The clinical success rate and PRAE incidence were 85.1% and 22.1%, respectively. Identified PRAEs included acute peritonitis (9.5%), fever (6.8%), abdominal pain (2.3%), and acute pancreatitis (1.4%). Multivariate analysis identified dilation device use (*p* = 0.01) and AGS (*p* = 0.03) as PRAE risk factors. AGS in patients who underwent fistula dilation (*p* = 0.02) and procedure time ≥41 min in those who underwent EUS‐HGS with AGS (*p* = 0.01) were PRAE risk factors.

**Conclusions:**

Fistula dilation and AGS are associated with an increased risk of PRAEs post‐EUS‐HGS. Careful postoperative follow‐up for such events is required in patients undergoing fistula dilation for EUS‐HGS with AGS and prolonged procedure time.

## Graphical abstract

This multicenter study identified use of dilation devices and antegrade stenting are risk factors for procedure‐related early adverse events following endoscopic ultrasound‐guided hepaticogastrostomy.

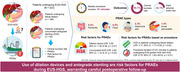

**Abbreviations**
AE: adverse event
AGS: antegrade stenting
ERCP: endoscopic retrograde cholangiopancreatography
EUS‐HGS: endoscopic ultrasound‐guided hepaticogastrostomy
MS: metal stent
PRAEs: procedure‐related early adverse events
PS: plastic stent
RBO: recurrent biliary obstruction
TRBO: time to recurrent biliary obstruction

## 1 Introduction

Endoscopic ultrasound‐guided hepaticogastrostomy (EUS‐HGS) is an effective procedure. [1] Notably, the gastrointestinal fistula created during EUS‐HGS facilitates biliary obstruction drainage in benign and malignant diseases [2, 3] and serves as an access route for biliary stone extraction [4, 5, 6].

While EUS‐HGS utility is characterized by its high success rate and reduction in re‐intervention, it carries a relatively high incidence of adverse events (AEs). Acute peritonitis, a common early AE, results from bile leakage through the fistula. Although minor bile leakage is inevitable, extensive leakage requires drainage to prevent severe abdominal infection. Bleeding at the puncture site and acute pancreatitis associated with concomitant antegrade stenting (AGS) also affect the clinical course [7]. Despite its clinical significance, research on procedure‐related early AEs (PRAEs) risk factors post‐EUS‐HGS remains limited. The identification of risk factors associated with PRAEs contributes to enhancing the safety of the procedure. Therefore, this study retrospectively evaluated patient backgrounds and procedure details to identify potential risk factors for PRAEs.

## 2 Methods

### 2.1 Study Population

**FIGURE 1:**
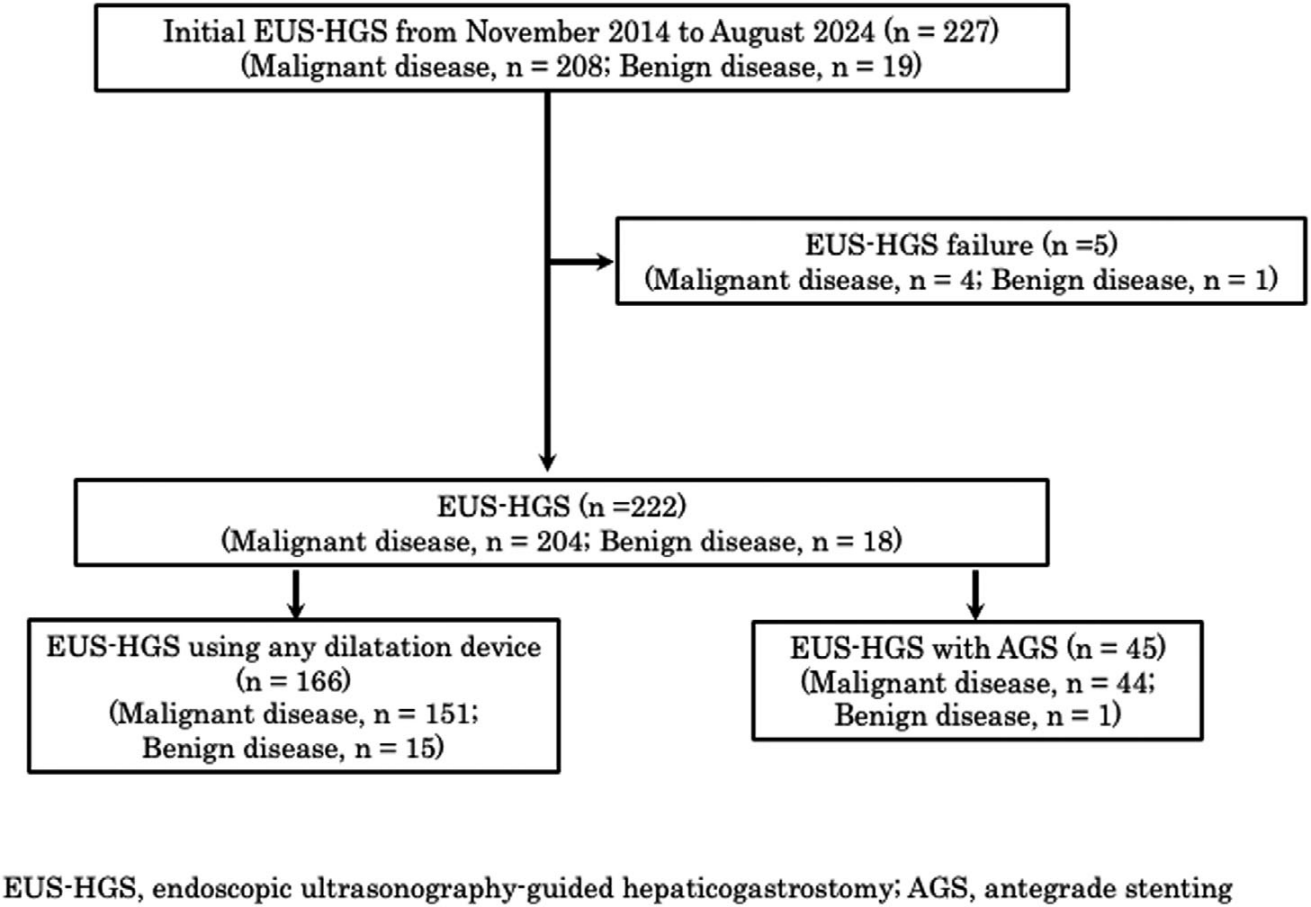
Flowchart of the study population selection., EUS‐HGS, endoscopic ultrasound‐guided hepaticogastrostomy; AGS, antegrade stenting.

This study included 222 patients and was conducted at a tertiary academic center and five medical hospitals in Kagoshima Prefecture, Japan, between November 2014 and August 2024 (Figure 1). Patients who underwent EUS‐HGS following failed or difficult endoscopic retrograde cholangiopancreatography (ERCP) were included. Clinical data were obtained from electronic medical records. All patients were followed up until death or December 31, 2024. Patients with technical failure in EUS‐HGS stenting were excluded (*n* = 5), including cases of difficult bile duct puncture (*n* = 3) and unsuccessful guidewire placement into the bile duct (*n* = 2).

### 2.2 EUS‐HGS Procedure

Patients fasted and received antibiotics before the procedure. Discontinuation of antithrombotic drugs was appropriately handled based on guidelines for endoscopic procedures with a high risk of bleeding. [8] All participating endoscopists had at least 10 years of experience, performing over 30 interventional EUS and 200 therapeutic ERCP cases annually. The appropriate intrahepatic bile duct, which was typically selected for B3, was punctured using an echoendoscope (GF‐UC240P or GF‐UCT260; Olympus Medical, Tokyo, Japan). After bile aspiration was performed to confirm the insertion of the puncture needle into the bile duct, a 0.025‐ or 0.018‐inch diameter guidewire was inserted into the bile duct. To advance the guidewire toward the hepatic hilum, a cannulation catheter (MTW, 5.5Fr, Dusseldorf, Germany; PR‐110Q, 3.5 Fr, Olympus Medical; uneven double‐lumen cannula, 6Fr, Piolax Medical Devices, Inc., Kanagawa, Japan) was inserted over it into the intrahepatic bile duct. If the cannulation catheter or stent delivery system could not pass directly through the bilio‐gastrointestinal fistula, dedicated dilation devices were used until the system could be advanced into the fistula. Subsequently, the stents were placed across the fistula. AGS was performed before fistula stenting if the guidewire reached the papilla of Vater. The dilation devices, stents, and concomitant AGS procedures were selected at the endoscopist's discretion. Table S1 presents the medical devices used for EUS‐HGS.

### 2.3 Outcome Measures

Clinical success was defined as a reduction in serum total bilirubin level to a normal level (<2.0 mg/dL) or less than half of the pretreatment level within 2 weeks [2]. Procedure time was defined as the duration from scope insertion to decannulation. Recurrent biliary obstruction (RBO) and time to RBO (TRBO) were defined as previously reported [2]. PRAEs were defined as AEs occurring within 2 weeks. AE definitions and severity were assessed based on established criteria for endoscopic AEs [9, 10]. Acute peritonitis was defined as new‐onset abdominal pain and fever with elevated inflammatory laboratory markers. Abdominal pain and fever (≥38°C) were defined as new onset of unknown origin within 24 h postoperatively.

### 2.4 Statistical Analyses

Data were analyzed using the chi‐square, Fisher's exact, or Mann–Whitney U test. TRBO was estimated using Kaplan–Meier curves and log‐rank tests. Since the PRAE incidence rate and risk factor were necessary for examining the case numbers, the number of PRAEs was counted by case number rather than by event. Multivariate analyses for PRAE risk factors were performed using stepwise logistic regression. All continuous variables in the multivariate analyses were set based on median values. Statistical significance was set at *p* < 0.05. All statistical analyses were performed using IBM SPSS Statistics for Windows, version 22 (IBM Corp., Armonk, NY, USA).

### 2.5 Ethics Statement

All patients provided written informed consent. This study's protocol was approved by the Institutional Review Board of Kagoshima University (Approval Number: 170218) and was conducted following the tenets of the Declaration of Helsinki and the Strengthening the Reporting of Observational Studies in Epidemiology guideline.

## 3 Results

### 3.1 Clinical Characteristics of Patients Who Underwent EUS‐HGS

Overall, 222 patients were enrolled (Table 1). Malignant diseases were present in 204 (91.9%) patients. Pancreatic cancer was observed in 89 (40.1%) patients, while 87 (39.2%) had biliary diseases, including biliary cancer, biliary stones, postoperative biliary obstruction, and pembrolizumab‐related sclerosing cholangitis in 71 (32.0%), 10 (4.5%), five (2.3%), and one (0.5%), respectively. The median total bilirubin level before EUS‐HGS was 6.3 (range: 0.3–33.7) mg/dL.

**TABLE 1:** Clinical characteristics of patients who underwent endoscopic ultrasound‐guided hepaticogastrostomy (EUS‐HGS).

	*n* = 222	Value
Age (years), median (range)		73 (37–93)
Male sex, *n* (%)		120 (54.1)
Malignant diseases, *n* (%)		204 (91.9)
Etiology of biliary obstruction, *n* (%)	Pancreatic diseases	89 (40.1)
	Biliary diseases	87 (39.2)
	Others	46 (20.7)
Reasons for EUS‐HGS, *n* (%)	Inaccessible papilla or ileobiliary anastomosis	94 (42.3)
	Difficult biliary cannulation	51 (23.0)
	Altered surgical anatomy	47 (21.2)
	Accessible papilla but inaccessible target bile duct	30 (13.5)
Serum level of total bilirubin (mg/dL), median (range)		6.3 (0.3–33.7)
Number of WBCs (/µL), median (range)		6845 (1200–26620)
Serum CRP level (mg/dL), median (range)		3.9 (0.01–26.4)
Location of biliary obstruction, n (%)	Distal bile duct	144 (64.9)
	Perihilar bile duct	78 (35.1)
Presence of cholangitis, n (%)		101 (45.5)
Presence of ascites, n (%)		44 (19.8)
Previous biliary drainage before EUS‐HGS, n (%)		72 (32.4)

CRP, C‐reactive protein; EUS‐HGS, endoscopic ultrasound‐guided hepaticogastrostomy; WBCs, white blood cells.

### 3.2 EUS‐HGS Procedure Details

The median procedure time for EUS‐HGS was 41 (range: 11–173) min (Table 2). Fistula dilation was performed in 166 (74.8%) patients. Metal stents (MSs) and plastic stents (PSs) (n = 107, 48.2%) were used for EUS‐HGS in 107 (48.2%) and 115 (51.8%) patients, respectively. For all benign cases, a PS for EUS‐HGS was selected. Among patients who underwent EUS‐HGS with AGS (*n* = 45), PSs (*n* = 37) for EUS‐HGS were used more frequently than MSs (n = 8). All stents used for AGS had delivery system sizes ≤7 Fr.

**TABLE 2:** Endoscopic ultrasound‐guided hepaticogastrostomy (EUS‐HGS) procedure details.

		*n* = 222
Procedure time of EUS‐HGS, min (range)		41 (11–173)
Use of dedicated dilation devices, *n* (%)		166 (74.8)
Mechanical bougie catheter, *n* (%), size, *n* (%)		112 (50.5)
7 Fr		112 (50.5)
10 Fr		3 (1.5)
Balloon catheter, *n* (%), size, *n* (%)		51 (23.0)
3 mm		2 (0.9)
4 mm		45 (20.3)
>4 mm		4 (1.8)
Electric cautery catheter, *n* (%)		37 (16.7)
6 Fr		34 (15.3)
7 Fr		3 (1.4)
Use of two or more types of dilation devices, *n* (%)		33 (14.9)
Bougie+balloon		28 (12.6)
Bougie+cautery		2 (0.9)
Baloon+cautery		1 (0.5)
Bougie+balloon+cautery		2 (0.9)
Used stent for EUS‐HGS		
Metal stent, *n* (%)		107 (48.2)
Partially covered	8 mm	25 (11.3)
	10 mm	65 (29.3)
Fully covered	6 mm	4 (1.8)
	8 mm	9 (4.1)
	10 mm	4 (1.8)
Plastic stent, *n* (%)		115 (51.8)
Single pigtail	7 Fr	103 (46.4)
	8 Fr	9 (4.1)
Straight	7 Fr	3 (1.4)
Concomitant antegrade stenting, *n* (%)		45 (20.3)
Metal stent, *n* (%)		43 (19.4)
Uncovered	8 mm	1 (0.5)
	10 mm	41 (18.5)
Fully covered	8 mm	1 (0.5)
Plastic stent, *n* (%)		2 (0.9)
Double pigtail	7 Fr	1 (0.5)
Straight	7 Fr	1 (0.5)

EUS‐HGS, endoscopic ultrasound‐guided hepaticogastrostomy.

Fistula dilatation was less frequently performed with stent delivery systems ≤7 Fr (n = 118; MS, *n* = 12; PS, *n* = 106) than with those >7 Fr (n = 104; MS, n = 95; PS, n = 9) (13.5% vs. 86.5%, *p* < 0.001). When comparing MS and PS used for EUS‐HGS, dilation devices were used more frequently in cases with MS than in those with PS (85.2% vs. 64.9%, *p* = 0.001). Balloon catheters (38.0% vs. 8.8%, *p* < 0.001) and multiple dilation devices (23.1% vs. 7.0%, *p* = 0.001) were used more frequently with MS than with PS for HGS. Mechanical bougie (52.8% vs. 48.2%, *p* = 0.50) and electric cautery (19.4% vs. 14.0%, *p* = 0.28) catheters were similar in both stent types.

### 3.3 Clinical Outcomes of EUS‐HGS

The clinical success rate was 85.1% (Table 3). PRAEs occurred in 49 (22.1%) patients, including acute peritonitis in 21 (9.5%); fever in 15 (6.8%); abdominal pain in five (2.3%); acute pancreatitis in three (1.4%); bleeding in two (1.0%); and acute cholecystitis, aspiration pneumonia, and pneumomediastinum in one (0.5%) each. No patient experienced two PRAEs simultaneously. The grades of PRAEs were mild, moderate, and severe in 37 (16.7%), 10 (4.5%), and two (0.9%) patients, respectively. Among patients with acute peritonitis, 15 (6.8%), four (1.8%), and two (0.9%) were in the mild, moderate, and severe categories, respectively. Specifically, among patients with moderate peritonitis, one underwent percutaneous aspiration of bile leakage in the abdominal cavity, another had EUS‐guided aspiration of the leakage, and a third required the placement of a fully covered MS for the uncovered part of the MS displaced into the fistula. One patient with severe peritonitis underwent additional AGS for distal biliary obstruction and percutaneous drainage, while those with other peritonitis cases were treated conservatively. Acute pancreatitis occurred in three (6.7%) patients who underwent AGS using an uncovered MS for the pancreatic cancer‐induced distal biliary obstruction and were treated conservatively. Acute cholecystitis caused by complete cystic duct obstruction due to tumor invasion requires percutaneous gallbladder aspiration. Among the two bleeding cases, one required transfusion. Minor bleeding was observed at the puncture site during the procedure; however, no active bleeding was detected on postoperative endoscopy or contrast‐enhanced computed tomography. The other case required endoscopic hemostasis and transfusion for a gastric ulcer caused by contact with the MS, identified 7 days post‐EUS‐HGS. Other PRAEs were treated conservatively.

**TABLE 3:** Clinical outcomes of the patients who underwent endoscopic ultrasound‐guided hepaticogastrostomy (EUS‐HGS).

	*n* = 222	
Clinical success, *n* (%)	189 (85.1)	
Procedure‐related early adverse events, *n* (%), grade, *n*	49 (22.1)	mild 37, moderate 10, severe 2
Acute peritonitis	21 (9.5)	mild 15, moderate 4, severe 2
Fever	15 (6.8)	mild 14, moderate 1
Abdominal pain	5 (2.3)	mild 5
Acute pancreatitis	3 (1.4)	mild 2, moderate 1
Bleeding	2 (1.0)	moderate 2
Acute cholecystitis	1 (0.5)	moderate 1
Aspiration pneumonia	1 (0.5)	moderate 1
Pneumomediastinum	1 (0.5)	mild 1
Event of RBO, n (%)	51 (23.1)	
TRBO (days), median (95% CI)	210 (123.79–296.21)	
OS in the malignant cases (*n* = 204), median (95%CI)	104 (84.11–123.89)	

CI, confidence interval; EUS‐HGS, endoscopic ultrasonography‐guided hepaticogastrostomy; OS, Overall survival; RBO, recurrent biliary obstruction; TRBO, time to recurrent biliary obstruction.

RBO occurred in 51 (23.1%) patients due to stent occlusion in 42 (18.9%) (MS, *n* = 12, 5.4%; PS, *n* = 30, 13.5%) and stent dislodgement in nine (4.1%) (MS, *n* = 7, 3.2%; PS, *n* = 2, 0.9%). The median (95% confidence interval [CI]) TRBO for MSs across the fistula (313 days, 95% CI = 154.01–474.99) was significantly longer than that for PSs (162 days, 95% CI = 85.80–238.19) (*p* = 0.02) (Figure S1a). Additionally, the median TRBO was similar between patients with and without PRAEs (313 vs. 210 days, *p* = 0.92) (Figure S1b) and between those with and without AGS (201 vs. 210 days, *p* = 0.66).

### 3.4 Risk Factors for PRAEs Post‐EUS‐HGS

Table 4 presents univariate and multivariate analyses of risk factors for PRAEs post‐EUS‐HGS. Multivariate analysis revealed the use of dilation devices (*p* = 0.01; odds ratio [OR] = 3.55; 95% CI = 1.35–9.36) and concomitant AGS (*p* = 0.03; OR = 2.62; 95% CI = 1.12–6.12) as risk factors for PRAEs.

**TABLE 4:** Univariate and multivariate analyses of the risk factors for procedure‐related early adverse events (AEs) after endoscopic ultrasound‐guided hepaticogastrostomy (EUS‐HGS).

	PRAEs		Univariate analysis	Multivariate analysis	
	(‐)	(+)			
	n = 173	n = 49	*p*‐Value	*p*‐Value	OR (95%CI)
Age <73 years, *n* (%)	93 (52.5)	31 (63.3)	0.24		
Male sex, *n* (%)	95 (54.9)	25 (51.0)	0.63		
Malignant diseases	160 (92.5)	44 (89.8)	0.36		
Etiology of biliary obstruction: Pancreatobiliary diseases, *n* (%)	133 (76.9)	42 (85.7)	0.18		
Reasons for EUS‐HGS: Inaccessible papilla or ileobiliary anastomosis, *n* (%)	110 (63.6)	34 (69.4)	0.45		
Serum level of total bilirubin ≥6.3 mg/dL, *n* (%)	85 (49.1)	26 (53.1)	0.63		
White blood cell count ≥6845 /µL, *n* (%)	86 (49.7)	25 (51.0)	0.87		
Serum level of CRP ≥3.9 mg/dL, *n* (%)	87 (50.3)	24 (49.0)	0.87		
Obstruction of distal bile duct, *n* (%)	110 (63.6)	34 (69.4)	0.45		
Presence of cholangitis, *n* (%)	77 (44.5)	24 (49.0)	0.58		
Presence of ascites, *n* (%)	37 (21.4)	7 (14.3)	0.27		
Biliary drainage before EUS‐HGS, *n* (%)	56 (32.4)	16 (32.7)	0.97		
19G puncture needle used for the procedure	171 (98.8)	48 (98.0)	0.53		
Concomitant antegrade stenting	32 (18.5)	13 (26.5)	0.22	0.03	2.62 (1.12–6.12)
Plastic stent placed across the fistula, *n* (%)	92 (53.2)	22 (44.9)	0.31		
Use of any dilation device	124 (71.7)	42 (85.7)	0.046	0.01	3.55 (1.35–9.36)
Procedure time ≥41 min, *n* (%)	82 (47.4)	30 (61.2)	0.09		

AEs, adverse events; CI, confidence interval; CRP, C‐reactive protein; EUS‐HGS, Endoscopic ultrasonography‐guided hepaticogastrostomy; OR, odds ratio; PRAEs, procedure‐related early adverse events.

Dilation devices were used more frequently in cases involving acute peritonitis than in those without peritonitis (100% vs. 72.1%, *p* < 0.01). Among dilation devices, mechanical bougie catheters (63.3% vs. 46.8%, *p* = 0.04), balloon catheters (34.7% vs. 19.7%, *p* = 0.03), and multiple dilation devices (24.5% vs. 12.1%, *p* = 0.03) were used more frequently in cases with PRAEs than in those without. Electric cautery catheter use (12.2% vs. 17.9%, *p* = 0.35) was similar between cases with and without PRAEs.

Cases with AGS had a lower presence of ascites (8.9% vs. 22.6%, *p* = 0.04), less previous biliary drainage before EUS‐HGS (20.0% vs. 35.6%, *p* = 0.05), less dilation device use (42.2% vs. 83.1%, *p* < 0.01), and more PS use across the fistula (82.2% vs. 43.5%, *p* < 0.01) than those without AGS. The median procedure time was similar between patients who underwent EUS‐HGS with and without AGS (42 vs. 37 min, *p* = 0.29).

### 3.5 Risk Factors for PRAEs in Patients Who Underwent Fistula Dilation for EUS‐HGS or Concomitant AGS

PRAE incidence was 25.3% (42/166) among patients who underwent fistula dilation for EUS‐HGS. Multivariate analysis showed that AGS (*p* = 0.02; OR = 3.11; 95% CI = 1.17–8.29) was associated with PRAE risk (Table 5). Among the PRAEs, acute peritonitis incidence was similar between cases with and without AGS (21.1% vs. 11.6%; *p* = 0.20). Mechanical bougie catheters (100% vs. 63.3%, *p* < 0.01) and PS for HGS (73.7% vs. 40.8%, *p* = 0.01) were used more frequently, while electric cautery catheters (0% vs. 25.2%, *p* = 0.01) were used less frequently in cases with AGS than in those without. Using balloon catheters (15.8% vs. 32.7%, *p* = 0.14), multiple dilation devices (15.8% vs. 20.4%, *p* = 0.45), and the median procedure time (48 vs. 42 min, *p* = 0.14) were similar between the two cases.

**TABLE 5:** Univariate and multivariate analyses of risk factors for procedure‐related early adverse events (AEs) in patients who underwent fistula dilation

	PRAEs		Univariate analysis	Multivariate analysis	
	(‐)	(+)			
	*n* = 124	*n* = 42	*p*‐Value	*p*‐Value	OR (95%CI)
Age <73 years, *n* (%)	63 (50.8)	28 (66.7)	0.07	0.15	0.57 (0.27–1.22)
Male sex, *n* (%)	70 (56.5)	23 (54.8)	0.85		
Malignant diseases	114 (91.9)	37 (88.1)	0.32		
Etiology of biliary obstruction: Pancreatobiliary diseases, *n* (%)	96 (77.4)	35 (83.3)	0.42		
Reasons for EUS‐HGS: Inaccessible papilla or ileobiliary anastomosis, *n* (%)	83 (66.9)	31 (73.8)	0.41		
Serum level of total bilirubin ≥6.3 mg/dL, *n* (%)	58 (46.8)	20 (47.6)	0.92		
White blood cell count ≥6845 /µL, *n* (%)	60 (48.4)	20 (47.6)	0.93		
Serum level of CRP ≥3.9 mg/dL, *n* (%)	62 (50.0)	21 (50.0)	>0.99		
Obstruction of distal bile duct, *n* (%)	79 (63.7)	29 (69.0)	0.53		
Presence of cholangitis, *n* (%)	54 (43.5)	22 (52.4)	0.32		
Presence of ascites, *n* (%)	29 (23.4)	6 (14.3)	0.21		
Biliary drainage before EUS‐HGS, *n* (%)	39 (31.5)	14 (33.3)	0.82		
19G puncture needle used for the procedure	122 (98.4)	41 (97.6)	0.59		
Concomitant antegrade stenting	10 (8.1)	9 (21.4)	0.02	0.02	3.11 (1.17–8.29)
Plastic stent placed across the fistula, *n* (%)	56 (45.2)	18 (42.9)	0.80		
Use of a mechanical bougie dilator	81 (65.3)	31 (73.8)	0.31		
Use of balloon dilator	34 (27.4)	17 (40.5)	0.11		
Use of an electric cautery dilator	31 (25.0)	6 (14.3)	0.15		
Use of two or more types of dilation devices	21 (16.9)	12 (28.6)	0.10		
Procedure time ≥41 min, *n* (%)	63 (50.8)	28 (66.7)	0.07		

AEs, adverse events; CI, confidence interval; CRP, C‐reactive protein; EUS‐HGS, endoscopic ultrasonography‐guided hepaticogastrostomy; OR, odds ratio; PRAEs, procedure‐related early adverse events.

PRAE incidence was 28.9% (13/45) in patients who underwent EUS‐HGS with AGS. Multivariate analysis revealed that procedure time ≥41 min (*p* = 0.01; OR = 6.75; 95% CI = 1.63–28.03) was associated with PRAE risk (Table 6). The median procedure time (46 vs. 33 min, *p* = 0.02) was significantly longer in cases with PRAEs than in those without. Among the PRAEs, acute peritonitis incidence was more frequent in cases with a procedure time ≥41 min than in those with a procedure time <41 min (23.5% vs. 0%; *p* = 0.016). Mechanical bougie catheters (69.2% vs. 31.3%, *p* = 0.02) were used more frequently in cases with PRAEs than in those without. Using balloon catheters (15.4% vs. 3.1%, *p* = 0.20), multiple dilation devices (15.8% vs. 3.1%, *p* = 0.20), and PS for HGS (15.4% vs. 18.8%, *p* = 0.58) were similar between the two cases.

**TABLE 6:** Univariate and multivariate analyses of risk factors for procedure‐related adverse events (AEs) in patients who underwent endoscopic ultrasonography‐guided hepaticogastrostomy (EUS‐HGS) with antegrade stenting (AGS).

	PRAEs		Univariate analysis	Multivariate analysis	
	(‐)	(+)			
	*n* = 32	*n* = 13	*p‐*Value	*p*‐Value	OR (95%CI)
Age <73 years, *n* (%)	13 (40.6)	6 (46.2)	0.73		
Male sex, *n* (%)	15 (46.9)	6 (46.2)	0.97		
Malignant diseases	32 (100.0)	12 (92.3)	0.29		
Etiology of biliary obstruction: Pancreatobiliary diseases, *n* (%)	23 (71.9)	13 (100.0)	0.03		
Reasons for EUS‐HGS: Inaccessible papilla or ileobiliary anastomosis, *n* (%)	22 (68.8)	9 (69.2)	0.63		
Serum level of total bilirubin ≥6.3 mg/dL, *n* (%)	14 (43.8)	7 (53.8)	0.54		
White blood cell count ≥6845 /µL, n (%)	13 (40.6)	4 (30.8)	0.40		
Serum level of CRP ≥3.9 mg/dL, *n* (%)	15 (46.9)	8 (61.5)	0.37		
Obstruction of distal bile duct, *n* (%)	24 (75.0)	8 (61.5)	0.29		
Presence of cholangitis, *n* (%)	11 (34.4)	4 (30.8)	0.55		
Presence of ascites, *n* (%)	3 (9.4)	1 (7.7)	0.67		
Biliary drainage before EUS‐HGS, *n* (%)	6 (18.8)	3 (23.1)	0.52		
19G puncture needle used for the procedure	32 (100.0)	13 (100.0)	>0.99		
Plastic stent placed across the fistula, *n* (%)	26 (81.3)	11 (84.6)	0.58		
Use of any dilation device	10 (31.3)	9 (69.2)	0.02		
Procedure time ≥41 min, *n* (%)	8 (25.0)	9 (69.2)	0.01	0.01	6.75 (1.63–28.03)

AEs, adverse events; AGS, antegrade stenting; CI, confidence interval; CRP, C‐reactive protein; EUS‐HGS, endoscopic ultrasonography‐guided hepaticogastrostomy; OR, odds ratio; PRAEs, procedure‐related early adverse events.

## 4 Discussion

This is the first multicenter study identifying fistula dilation and AGS as risk factors for PRAEs. Concomitant AGS and a prolonged procedure time were risk factors in patients who underwent fistula dilation for EUS‐HGS and those who underwent EUS‐HGS with AGS, respectively.

PRAE incidence post‐EUS‐HGS was 17.5–29.0% [1, 11, 12]. Among PRAEs, acute peritonitis (including abdominal pain), cholangitis (including sepsis, bacteremia, and fever), and bleeding were reported at rates of 1.1%–24%, 2.1%–24%, and 0.9–1.8%, respectively [13]. A recent meta‐analysis found that bile leakage accounted for 30% of early AEs [14, 15]. The primary cause of PRAEs—including acute peritonitis, abdominal pain, and fever—was bile leakage through the fistula.

Several risk factors for PRAEs post‐EUS‐HGS have recently been linked to bile leakage. A puncture distance <2.5 cm between the gastrointestinal tract and liver parenchyma was identified as a factor [16]. After puncturing the intrahepatic duct, aspiration of bile juice >10 mL reduced PRAE occurrence [17]. Three single‐center retrospective studies revealed fistula dilation as a risk factor for PRAEs [15, 18, 19]. Fistula dilation of EUS‐HGS may be one of the universal risk factors for PRAEs since this study included heterogeneous factors, including benign and malignant diseases and PS, and MS.

The stent delivery system's small diameter enables stent placement without fistula dilation [15, 20]. However, not all procedures can be performed without fistula dilation. A thinner fistula prevents the device advancement into the bile duct, and the acute angle between the bile duct and the puncture needle further hinders device insertion [15, 21]. Fistula dilation helps advance the devices. In this study, 74.8% of the patients underwent a dilation procedure, especially those using a stent delivery system >7 Fr was used for EUS‐HGS. The development of thinner stent delivery systems that do not require dilatation is anticipated.

No reports have identified AGS post‐EUS‐HGS as a PRAE risk, while AGS is expected to reduce bile leakage into the abdominal cavity by facilitating bile extraction into the intestine. In patients who underwent EUS‐HGS with AGS, easily replaceable PSs are usually placed across the fistula to ensure access to the bile duct. Khashab et al. reported that PS was a risk factor for PRAEs [22]. Previous studies showed that EUS‐HGS with AGS tends to have more prolonged procedure times and a higher PRAE incidence than EUS‐HGS alone [23, 24]. Prolonged procedure time for EUS‐HGS with AGS may result in more bile leakage into the abdominal cavity, as acute peritonitis incidence was high in cases that required a prolonged procedure time for EUS‐HGS with AGS in the study. AGS causes acute pancreatitis by affecting the pancreatic ducts, with a 4.9–16.0% incidence rate [23, 24, 25, 26]. The higher incidence of acute pancreatitis due to AGS may explain the increase in PRAEs since all pancreatitis cases occurred in patients who underwent AGS, while acute peritonitis was similar in those who underwent fistula dilatation in the study. Additionally, the complexity of EUS‐HGS with AGS may explain the causes of PRAEs. In this study, TRBO showed similar results for EUS‐HGS with and without AGS. Therefore, the use of AGS is not required in all cases. Safely completing EUS‐HGS without AGS is preferable rather than prolonging the procedure with concomitant AGS.

Bleeding was mainly caused by vascular injury during the puncture and dilation procedures [7] and by the ulcer formation due to stent contact [24]. Consequently, clinical symptoms and blood tests are important for early detection of bleeding.

This study had some limitations. First, it was a retrospective, multicenter study conducted in institutions with heterogeneous technical levels of endoscopists, medical equipment, and devices. In the study, the endoscopist's experience with EUS‐HGS was not considered for the analyses of the risk factors of PRAEs because several endoscopists performed the procedures at multiple facilities. The experience of the endoscopists may affect the incidence of PRAEs. Second, the procedural techniques were not specified before the study, and the choice of devices and AGS implementation was at the operator's discretion. While the amount of bile aspirated after puncture was small, it was not measured and varied between procedures. EUS‐HGS with AGS without fistula dilation is a relatively recent procedure enabled by thinner stent delivery systems development. The potential confounding factors cannot be eliminated due to the retrospective study design. Therefore, the effect of the use of dilation devices and concomitant AGS on PRAE incidence should be further evaluated in randomized controlled trials. Third, the strict classification of PRAEs was challenging. For instance, differentiating between cases of fever that were indicative of acute peritonitis, non‐occlusive cholangitis, or aspiration pneumonia could be challenging. Nevertheless, this study provides valuable insights into managing patients undergoing EUS‐HGS, especially since EUS‐HGS has not yet been fully established, dilation devices are still required in many cases, and the clinical outcomes of concomitant AGS remain debatable.

In conclusion, the use of dilation devices and AGS is associated with increased PRAE risk post‐EUS‐HGS. Therefore, careful follow‐up is required for patients undergoing fistula dilation, concomitant AGS after the dilation, and prolonged procedure time for AGS. Further advancement in thinner delivery systems for dedicated stents is expected to reduce PRAE incidence and procedure time.

## Author Contributions

**Shinichi Hashimoto**: Conceptualization; data curation; formal analysis; funding acquisition; investigation; methodology; project administration; supervision; validation; visualization; writing ‐ original draft. **Hiroki Taguchi**: Data curation; investigation; methodology; project administration; resources. **Norimasa Araki**: Data curation; investigation; resources. **Yu Yamazato**: Data curation; investigation; resources. **Hiroki Iwata**: Data curation; investigation; resources. **Yuji Tabira**: Data curation; investigation; resources. **Ryusuke Shibata**: Data curation; investigation; resources. **YUSUKE KAMIKIHARA**: Data curation; investigation; resources. **Koshiro Toyodome**: Data curation; investigation; resources. **Issei Kojima**: Data curation; investigation; methodology. **Takafumi Hamada**: Data curation; investigation. **Kengo Tsuneyoshi**: Data curation; investigation. **Yoshitaka Nakamura**: Data curation; investigation; methodology. **Makoto Hinokuchi**: Data curation; investigation; methodology. **Shiho Arima**: Data curation; investigation. **Shiroh Tanoue**: Data curation; formal analysis; investigation; methodology; writing ‐ review and editing. **Fumisato Sasaki**: Supervision; validation; writing ‐ review and editing. **Shuji Kanmura**: Writing ‐ review and editing. **Akio Ido**: Project administration; writing ‐ review and editing.

## Ethics Statement

**Approval of the research protocol by an Institutional Review Board**: The study protocol was approved by the Institutional Review Board of Kagoshima University (Approval Number: 170218). This study adhered to the tenets of the Declaration of Helsinki and followed the Strengthening the Reporting of Observational Studies in Epidemiology (STROBE) checklist.

## Consent

Written informed consent for the procedure was obtained from all the patients.

## Conflicts of Interest

The authors declare no conflicts of interest.

## Clinical Trial Registration

N/A.

## Supporting information




**TABLE S1** Devices used for EUS‐HGS. EUS‐HGS, endoscopic ultrasound‐guided hepaticogastrostomy

**FIGURE S1** Kaplan–Meier curves for time to recurrent biliary obstruction (TRBO). (a) TRBO with the metal stent (solid line; 313 days; 95% CI = 151.01–474.99) was significantly longer than that with the plastic stent (dotted line; 162 days; 95% CI = 85.81–238.19) (*p* = 0.02). (b) TRBO between the cases with procedure‐related adverse events (solid line; *n* = 49, 313 days; 95% CI = 121.94–504.06) and those without (dotted line; *n* = 173, 210 days; 95% CI = 124.54–295.46) were similar (*p* = 0.92)., 95% CI, 95% confidence interval

**Supporting File 3**. deo270211‐sup‐0003‐FigureS1B.tif

## References

[1] H. Isayama , Y. Nakai , T. Itoi , et al., “Clinical Practice Guidelines for Safe Performance of Endoscopic Ultrasound/Ultrasonography‐Guided Biliary Drainage: 2018,” Journal of Hepato‐Biliary‐Pancreatic Sciences 26, no. 7 (2019): 249–269, 10.1002/jhbp.631. 31025816 PMC7064894

[2] S. Hashimoto , Y. Iwashita , H. Taguchi , et al., “Comparison of Recurrent Biliary Obstruction With the Use of Metal and Plastic Stents in EUS‐Guided Biliary Drainage: A Propensity Score‐Matched Analysis,” Endoscopic Ultrasound 12, no. 1 (2023): 64–73, 10.4103/EUS-D-21-00251. 36510868 PMC10134919

[3] F. Caillol , S. Godat , A. Solovyev , et al., “EUS‐BD for Calibration of Benign Stenosis of the Bile Duct in Patients With Altered Anatomy or Inaccessible Papilla,” Endoscopy International Open 12, no. 3 (2024): E377–E384, 10.1055/a-2261-2968. 38464978 PMC10919993

[4] S. Hashimoto , T. Fujita , and A. Ido , “Emergent Endoscopic Ultrasonography‐Guided Antegrade Treatment for Acute Biliary Pancreatitis in a Patient With Altered Gastrointestinal Anatomy,” Digestive Endoscopy 31, no. 3 (2019): e58–e59, 10.1111/den.13345. 30648320

[5] T. Iwashita , Y. Iwasa , A. Senju , et al., “Comparing Endoscopic Ultrasound‐Guided Antegrade Treatment and Balloon Endoscopy‐Assisted Endoscopic Retrograde Cholangiopancreatography in the Management of Bile Duct Stones in Patients With Surgically Altered Anatomy: A Retrospective Cohort Study,” Journal of Hepato‐Biliary‐Pancreatic Sciences 30, no. 8 (2023): 1078–1087, 10.1002/jhbp.1321. 36862054

[6] T. Sato , Y. Nakai , H. Kogure , et al., “ERCP Using Balloon‐Assisted Endoscopes versus EUS‐Guided Treatment for Common Bile Duct Stones in Roux‐en‐Y Gastrectomy,” Gastrointestinal Endoscopy 99, no. 2 (2024): 193–203.e5, 10.1016/j.gie.2023.09.001. 37709151

[7] H. Ishiwatari , H. Sakamoto , T. Doi , and M. Yamamura , “Prevention of Adverse Events in Endoscopic Ultrasound‐Guided Biliary Drainage,” DEN Open 6, no. 1 (2025): e70145, 10.1002/deo2.70145. 40416588 PMC12098953

[8] M. Kato , N. Uedo , S. Hokimoto , et al., “Guidelines for Gastroenterological Endoscopy in Patients Undergoing Antithrombotic Treatment: 2017 Appendix on Anticoagulants Including Direct Oral Anticoagulants,” Digestive Endoscopy 30, no. 4 (2018): 433–440, 10.1111/den.13184. 29733468

[9] P. B. Cotton , G. M. Eisen , L. Aabakken , et al., “A Lexicon for Endoscopic Adverse Events: Report of an ASGE Workshop,” Gastrointestinal Endoscopy 71, no. 3 (2010): 446–454, 10.1016/j.gie.2009.10.027. 20189503

[10] H. Isayama , T. Hamada , T. Fujisawa , et al., “TOKYO Criteria 2024 for the Assessment of Clinical Outcomes of Endoscopic Biliary Drainage,” Digestive Endoscopy 36, no. 11 (2024): 1195–1210, 10.1111/den.14825. 38845085

[11] J. Li , J. Tang , F. Liu , and J. Fang , “Comparison of Choledochoduodenostomy and Hepaticogastrostomy for EUS‐Guided Biliary Drainage: A Meta‐Analysis,” Frontiers in Surgery 9 (2022): 811005, 10.3389/fsurg.2022.811005. 35356500 PMC8959983

[12] S. W. van der Merwe , R. L. J. van Wanrooij , M. Bronswijk , et al., “Therapeutic Endoscopic Ultrasound: European Society of Gastrointestinal Endoscopy (ESGE) Guideline,” Endoscopy 54, no. 2 (2022): 185–205, 10.1055/a-1717-1391. 34937098

[13] S. Nakaji , H. Takahashi , W. Yoshioka , et al., “Risk Factors of Early Adverse Events Associated With Endoscopic Ultrasound‐Guided Hepaticogastrostomy Using Self‐Expandable Metal Stent,” Endoscopy International Open 12, no. 1 (2024): E164–E175, 10.1055/a-2240-1100. 38292592 PMC10827478

[14] K. Wang , J. Zhu , L. Xing , Y. Wang , Z. Jin , and Z. Li , “Assessment of Efficacy and Safety of EUS‐Guided Biliary Drainage: A Systematic Review,” Gastrointestinal Endoscopy 83, no. 6 (2016): 1218–1227, 10.1016/j.gie.2015.10.033. 26542374

[15] M. Itonaga , R. Ashida , T. Emori , et al., “Safety of Skipping the Tract Dilation Step for EUS‐Guided Biliary Drainage in Patients With Unresectable Malignant Biliary Obstruction (With Video),” Surgical Endoscopy 38, no. 4 (2024): 2288–2296, 10.1007/s00464-024-10731-z. 38488871

[16] Y. Yamamoto , T. Ogura , N. Nishioka , et al., “Risk Factors for Adverse Events Associated With Bile Leak During EUS‐Guided Hepaticogastrostomy,” Endoscopic Ultrasound 9, no. 2 (2020): 110–115, 10.4103/eus.eus_68_19. 32295968 PMC7279085

[17] H. Ishiwatari , T. Satoh , J. Sato , et al., “Bile Aspiration During Eus‐Guided Hepaticogastrostomy is Associated With Lower Risk of Postprocedural Adverse Events: A Retrospective Single‐Center Study,” Surgical Endoscopy 35, no. 12 (2021): 6836–6845, 10.1007/s00464-020-08189-w. 33398558

[18] Y. Fujii , H. Kato , H. Himei , et al., “Double Guidewire Technique Stabilization Procedure for Endoscopic Ultrasound‐Guided Hepaticogastrostomy Involving Modifying the Guidewire Angle at the Insertion Site,” Surgical Endoscopy 36, no. 12 (2022): 8981–8991, 10.1007/s00464-022-09350-3. 35927355

[19] A. Ohno , N. Fujimori , T. Kaku , et al., “Feasibility and Efficacy of Endoscopic Ultrasound‐Guided Hepaticogastrostomy Without Dilation: A Propensity Score Matching Analysis,” Digestive Diseases and Sciences 67 (2022): 5676–5684, 10.1007/s10620-022-07555-z. 35689110

[20] K. Takeshita , S. Hijioka , Y. Nagashio , et al., “Usefulness of a Laser‐Cut Covered Metal Stent With a 7F Delivery Sheath in Endoscopic Ultrasound‐Guided Biliary Drainage Without Fistula Dilation,” Endoscopy International Open 11, no. 1 (2023): E97–E104, 10.1055/a-1997-9149. 36712906 PMC9879640

[21] K. Bessho , T. Ogura , S. Ueno , et al., “Moving Scope Technique Improves Technical Success Rate of Device Insertion During EUS‐Guided Hepaticogastrostomy (With Video),” Therapeutic Advances in Gastroenterology 16 (2023): 17562848231207004, 10.1177/17562848231207004. 37900005 PMC10605674

[22] M. A. Khashab , A. A. Messallam , I. Penas , et al., “International Multicenter Comparative Trial of Transluminal EUS‐Guided Biliary Drainage via Hepatogastrostomy vs. Choledochoduodenostomy Approaches,” Endoscopy International Open 4, no. 2 (2016): E175–E181, 10.1055/s-0041-109083. 26878045 PMC4751013

[23] H. Ishiwatari , K. Ishikawa , F. Niiya , et al., “Endoscopic Ultrasound‐Guided Hepaticogastrostomy versus Hepaticogastrostomy With Antegrade Stenting for Malignant Distal Biliary Obstruction,” Journal of Hepato‐Biliary‐Pancreatic Sciences 29, no. 6 (2022): 703–712, 10.1002/jhbp.1118. 35094496

[24] A. Schoch , A. Lisotti , T. Walter , et al., “Efficacy of EUS‐Guided Hepaticogastrostomy in Prolonging Survival of Patients With Perihilar Cholangiocarcinoma,” Endoscopic Ultrasound 11, no. 6 (2022): 487–494, 10.4103/EUS-D-22-00014. 36537386 PMC9921975

[25] H. So , D. Oh , M. Takenaka , et al., “Initial Experience of Endoscopic Ultrasound‐Guided Antegrade Covered Stent Placement With Long Duodenal Extension for Malignant Distal Biliary Obstruction (With Video),” Journal of Hepato‐Biliary‐Pancreatic Sciences 28, no. 12 (2021): 1130–1137, 10.1002/jhbp.1011. 34118136 PMC9290461

[26] T. Iwashita , I. Yasuda , T. Mukai , et al., “Endoscopic Ultrasound‐Guided Antegrade Biliary Stenting for Unresectable Malignant Biliary Obstruction in Patients With Surgically Altered Anatomy: Single‐center Prospective Pilot Study,” Digestive Endoscopy 29, no. 3 (2017): 362–368, 10.1111/den.12800. 28066983

