# Supplementary material for: Dilation Device Use and Concomitant Antegrade Stenting are Associated With Procedure‐related Early Adverse Events After Endoscopic Ultrasound‐guided Hepaticogastrostomy: A Retrospective Multicenter Study
Source: DEN Open. 2025 Oct 7;6(1):e70211. doi: 10.1002/deo2.70211 (PMC12501838; doi:10.1002/deo2.70211)
Supplement: Supplementary file 1 — TABLE S1 Devices used for EUS‐HGS. EUS‐HGS, endoscopic ultrasound‐guided hepaticogastrostomy [file DEO2-6-e70211-s001.docx]

Supplementary Table. Devices used for EUS-HGS

| Devices | | Product name | Company | Delivery system size | Size of the dilation or stent diameter | n |
| --- | --- | --- | --- | --- | --- | --- |
| Dilation devices | |  |  |  |  |  |
|  | Bougie catheter | ES dilator | Zeon Medical Inc., Tokyo, Japan | 7 Fr | 7 Fr | 81 |
|  |  | Tornus ES | Olympus Medical, Tokyo, Japan | 7 Fr | 7 Fr | 31 |
|  |  | Multifunction catheter | Gadelius Medical Co., Tokyo, Japan | 10 Fr | 10 Fr | 3 |
|  | Balloon catheter | REN | Kaneka Medix Co., Osaka, Japan | 6.4 Fr | 3 mm | 2 |
|  |  |  |  | 6.4 Fr | 4 mm | 41 |
|  |  |  |  | 6.4 Fr | 8 mm | 2 |
|  |  |  |  | 6.4 Fr | 12 mm | 2 |
|  |  | Harricane | Boston Scientific Corp., Natick, MA, USA | 5.8 Fr | 4 mm | 3 |
|  |  | ZARA | Century Medical Inc., Tokyo, Japan | 6.3 Fr | 4 mm | 1 |
|  | Electric cautery dilator | Cyst-Gastro-Set | Medi-Globe GmbH, Rosenheim, Germany | 6 Fr | 6 Fr | 34 |
|  |  | Fine025 | Medico's Hirata Inc. Osaka, Japan | 7 Fr | 7 Fr | 3 |
| Used stent for EUS-HGS | |  |  |  |  |  |
|  | Partially covered metal stent | Niti‑S EUS-BD system | TaeWoong Medical, Seoul, South Korea | 8.5 Fr | 8 mm | 25 |
|  |  |  |  | 8.5 Fr | 10 mm | 65 |
|  | Fully covered metal stent | HANAROSTENT Benefit | Boston Scientific Corp., Natick, MA, USA | 5.9 Fr | 6 mm | 4 |
|  |  |  |  | 5.9 Fr | 8 mm | 8 |
|  |  | HANAROSTENT | Boston Scientific Corp., Natick, MA, USA | 8 Fr | 10 mm | 1 |
|  |  | ZEOSTENT | Zeon Medical Inc., Tokyo, Japan | 8.5 Fr | 10 mm | 3 |
|  |  | BONASTENT | Standard Sci Tech Inc., Seoul, South Korea | 8 Fr | 8 mm | 1 |
|  | Single pigtail-type plastic stent | Type IT | Gadelius Medical Co., Tokyo, Japan | 7 Fr | 7 Fr | 103 |
|  |  |  |  | 8 Fr | 8 Fr | 9 |
|  | Straight-type plastic stent | Flexima | Boston Scientific Corp., Natick, MA, USA | 7 Fr | 7 Fr | 1 |
|  |  | Advanix J | Boston Scientific Corp., Natick, MA, USA | 7 Fr | 7 Fr | 1 |
|  |  | SUZAKU | Kaneka Medix Co., Osaka, Japan | 7 Fr | 7 Fr | 1 |
| Used stent for AGS | |  |  |  |  |  |
|  | Uncovered metal stent | ZEOSTENT V | Zeon Medical Inc., Tokyo, Japan | 5.4 Fr | 8 mm | 1 |
|  |  |  |  | 5.4 Fr | 10 mm | 39 |
|  |  | Niti-S Large Cell SR Slim Delivery | TaeWoong Medical, Seoul, South Korea | 6 Fr | 10 mm | 2 |
|  | Fully covered metal stent | HANAROSTENT Benefit | Boston Scientific Corp., Natick, MA, USA | 5.9 Fr | 8 mm | 1 |
|  | Double pigtail-type plastic stent | Mediglobe biliary stent | Medi-Globe GmbH, Rosenheim, Germany | 7 Fr | 7 Fr | 1 |
|  | Straight-type plastic stent | SUZAKU | Kaneka Medix Co., Osaka, Japan | 7 Fr | 7 Fr | 1 |

EUS-HGS, endoscopic ultrasonography-guided hepaticogastrostomy; AGS, antegrade stenting
