# Supplementary figures and images for: Dilation Device Use and Concomitant Antegrade Stenting are Associated With Procedure‐related Early Adverse Events After Endoscopic Ultrasound‐guided Hepaticogastrostomy: A Retrospective Multicenter Study
Source: DEN Open. 2025 Oct 7;6(1):e70211. doi: 10.1002/deo2.70211 (PMC12501838; doi:10.1002/deo2.70211)

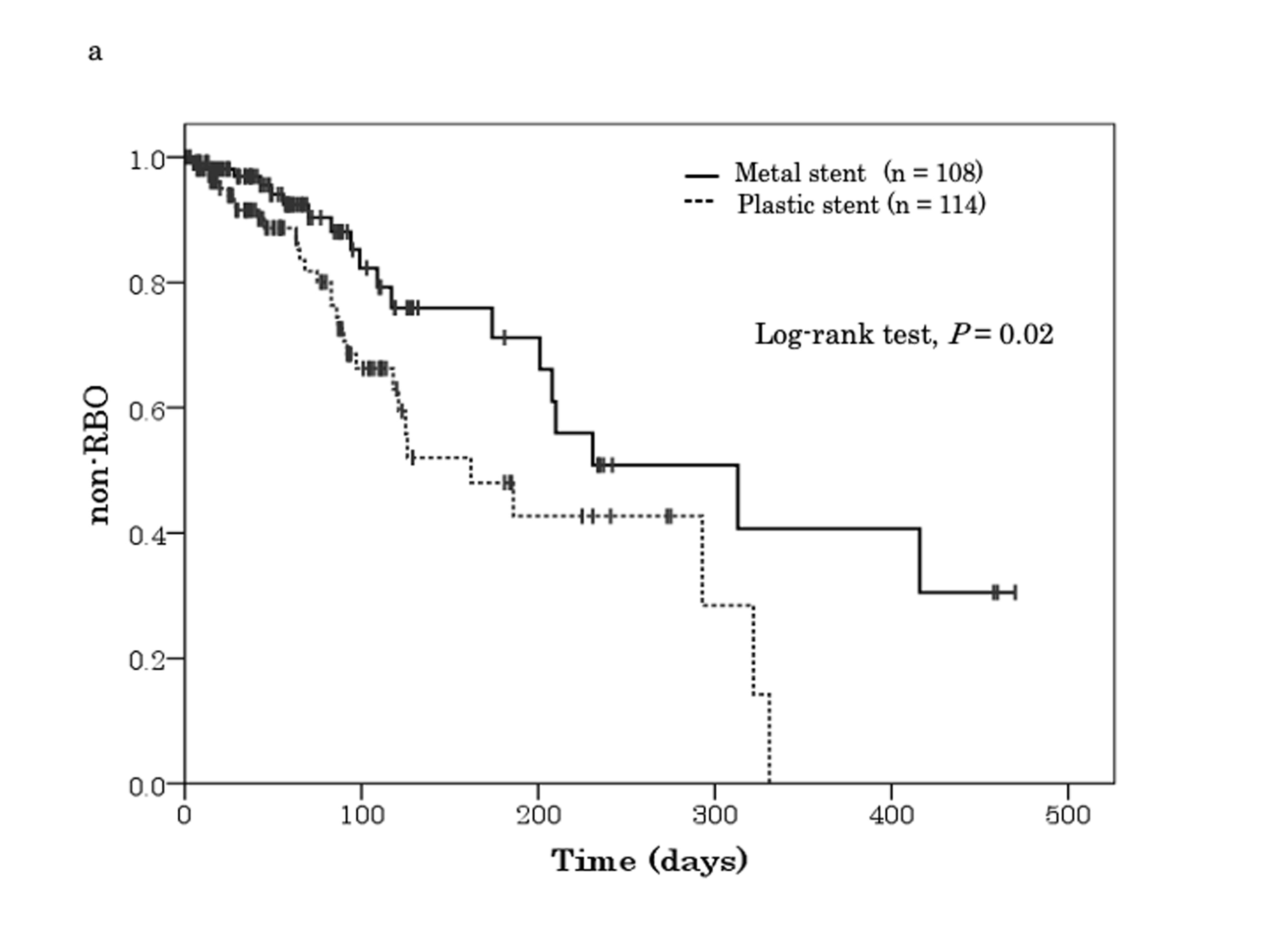

Supplement: Supplementary file 2 — FIGURE S1 Kaplan–Meier curves for time to recurrent biliary obstruction (TRBO). (a) TRBO with the metal stent (solid line; 313 days; 95% CI = 151.01–474.99) was significantly longer than that with the plastic stent (dotted line; 162 days; 95% CI = 85.81–238.19) (p = 0.02). (b) TRBO between the cases with procedure‐related adverse events (solid line; n = 49, 313 days; 95% CI = 121.94–504.06) and those without (dotted line; n = 173, 210 days; 95% CI = 124.54–295.46) were similar (p = 0.92)., 95% CI, 95% confidence interval [file DEO2-6-e70211-s002.tif]

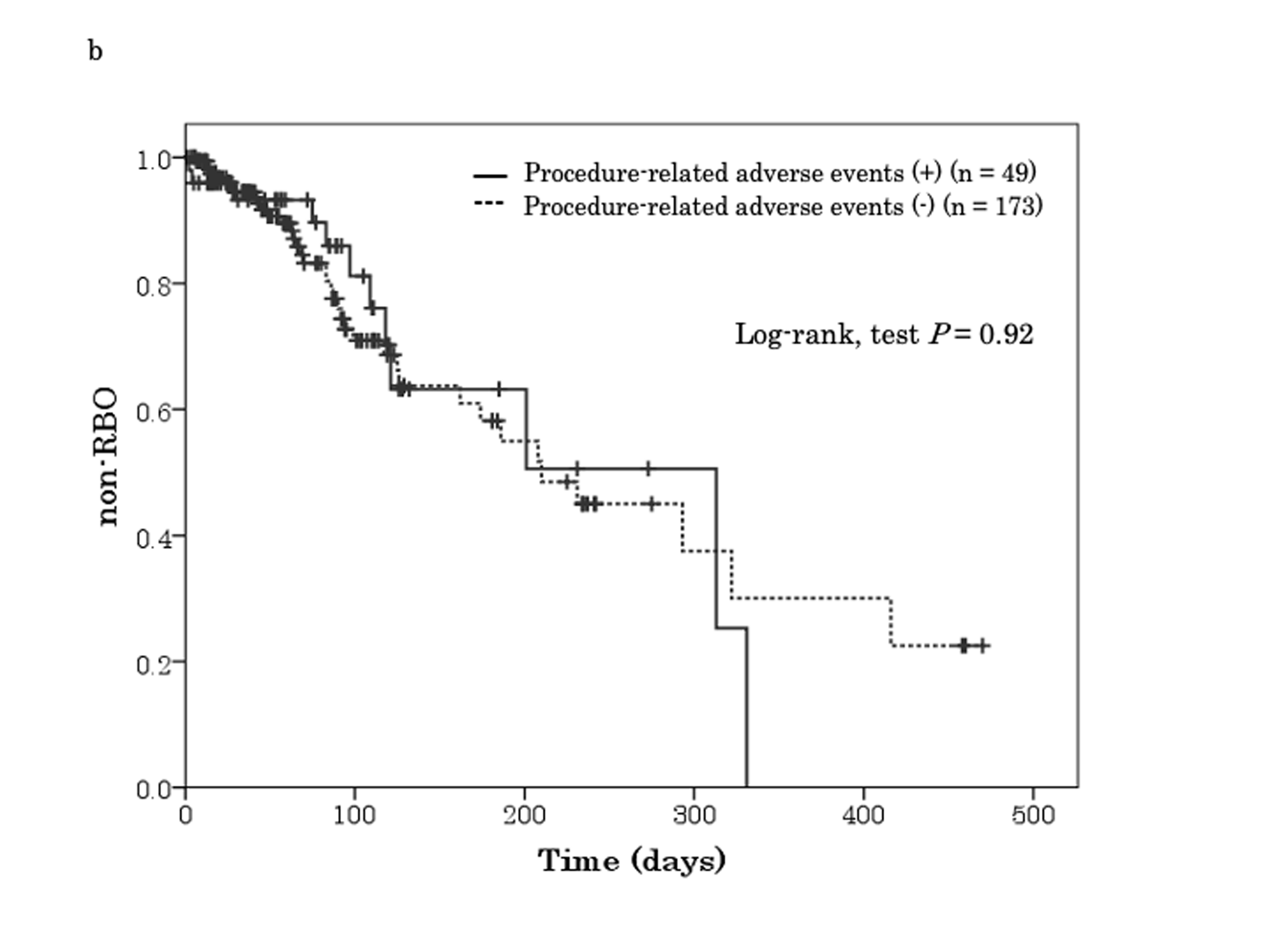

Supplement: Supplementary file 3 — Supporting File 3. deo270211‐sup‐0003‐FigureS1B.tif [file DEO2-6-e70211-s003.tif]
